# Increased rates of *bla*_NDM_ in *Pseudomonas aeruginosa* in a tertiary care hospital in southern Brazil

**DOI:** 10.1016/j.bjid.2025.104523

**Published:** 2025-04-10

**Authors:** Patricia Orlandi Barth, Dariane Castro Pereira, Camila Mörschbächer Wilhelm, Kellen Figueira Tragnago, Afonso Luís Barth

**Affiliations:** a Laboratório de Pesquisa em Resistência Bacteriana, Hospital de Clínicas de Porto Alegre, Porto Alegre, RS, Brazil; b Universidade Federal do Rio Grande do Sul, Programa de Pós-Graduação em Ciências Médicas (PPGCM/UFRGS), Porto Alegre, RS, Brazil

**Keywords:** Pseudomonas aeruginosa Carbapenemases NDM

## Abstract

Carbapenem-Resistant *Pseudomonas Aeruginosa* (CRPA) is considered as one of the high priority pathogens by the World Health Organization. As CRPA carbapenemase producers have increased worldwide, the aim of this study was to evaluate the carbapenemase prevalence in CRPA at a tertiary care hospital in Brazil”*.* All 395 CRPA identified in the period of September 2021 to May 2024 were evaluated by multiplex real-time polymerase chain reaction (qPCR-HRM) for the following carbapenemase genes: *bla*_KPC_, *bla*_NDM_, *bla*_OXA-48-like_, *bla*_IMP_, *bla*_VIM,_*bla*_SPM_ and *bla*_GES_. In the first period analyzed (September to December 2021), almost 70 % of the isolates were negative for the 7 tested genes, and the *bla*_NDM_ was found in 27.3 % of the CRPA. In the following semesters there was an increase of *bla*_NDM_ as follows: January to June of 2022 = 29.8 %; July to December of 2022 = 43.8 %; January to June of 2023 = 42.4 %; July to December 2023 = 58.9 % and January to May of 2024 = 59.5 % of *bla*_NDM_. The prevalence of the other carbapenemases remained low. These results indicated an important increase of the *bla*_NDM_ gene, overcoming the CRPA non-carbapenemase producers in our institution.

*Pseudomonas aeruginosa* is a non-fermenting gram-negative bacilli typically found in healthcare settings, usually associated with urinary tract, respiratory and bloodstream infections [1] Carbapenem-Resistant *P. Aeruginosa* (CRPA) is particularly worrying and is considered a high-priority pathogen in the 2024 Bacterial Priority Pathogens List due to their global threat and limited therapeutic options [2] Furthermore, CRPA are associated with worse morbidity and mortality outcomes compared to susceptible isolates, as well as an important impact on hospitalization costs [3,4]

The main mechanisms which lead to carbapenem resistance in *P. aeruginosa* are efflux pump overexpression, overproduction of AmpC beta-lactamase usually associated with porin loss or inactivation, and, more rarely, production of carbapenemases [5] While the most prevalent carbapenemase gene worldwide are *bla*_VIM_ followed by *bla*_GES_ [6] *bla*_NDM_ has been emerging in the last years in CRPA from Europe [7] Africa [8] and other countries [9]

Until 2010, the *bla*_SPM_ gene used to be the most common carbapenemase among CRPA in southern Brazil [10,11] However, a shift from *bla*_SPM_ to other carbapenemase genes, including *bla*_NDM_, was described in the last three years [12] The increasing rates of *bla*_NDM_ is highly alarming, as this gene is commonly associated with resistance to carbapenems and other antimicrobial classes[13,14] which limits treatment options. Therefore, the aim of this study was to evaluate carbapenemase prevalence in CRPA clinical isolates from a tertiary care hospital in southern Brazil.

All CRPA (only one isolate per patient) identified at the Microbiology Laboratory of Hospital de Clínicas de Porto Alegre during the period of September 2021 to May 2024 were evaluated for carbapenemase genes by multiplex real-time Polymerase Chain Reaction (qPCR) using High-Resolution Melting (HRM) analysis for *bla*_SPM_, *bla*_KPC_, *bla*_NDM_, *bla*_OXA-48-like_, *bla*_IMP_, *bla*_VIM_ and *bla*_GES_ as previously described [15] DNA was extracted by thermal lysis, and qPCR HRM was performed using QuantStudio-3® (Thermo Fisher). The identification of the isolates was performed by Matrix-Assisted Laser-Desorption Ionization Time-Of-Flight Mass Spectrometry ‒ MALDI-TOF-MS (Bruker Daltonics) and resistance to carbapenems was determined by meropenem disk diffusion method [16]

In the period of this study, 395 CRPA isolates of *P. aeruginosa* were evaluated. A total of 218 (55.2 %) presented carbapenemase genes by qPCR HRM assays. In the second semester of 2021, when carbapenemase evaluation in CRPA initiated, almost 70 % (38/55) of isolates were negative for the 7 genes tested, and *bla*_NDM_ gene was detected in 27.3 % (15/55). In the following semesters, there was a steadily increasing of *bla*_NDM_ as follows: January to June of 2022 = 29.8 % (17/57) of *bla*_NDM_, July to December of 2022 = 43.8 % (21/48); January to June of 2023 = 42.4 % (28/66); July to December 2023 = 58.9 % (53/90) and January to May of 2024 = 59.5 % (47/79) of *bla*_NDM_. The prevalence of the other carbapenemases remained constant (Fig. 1).

**Fig. 1:**
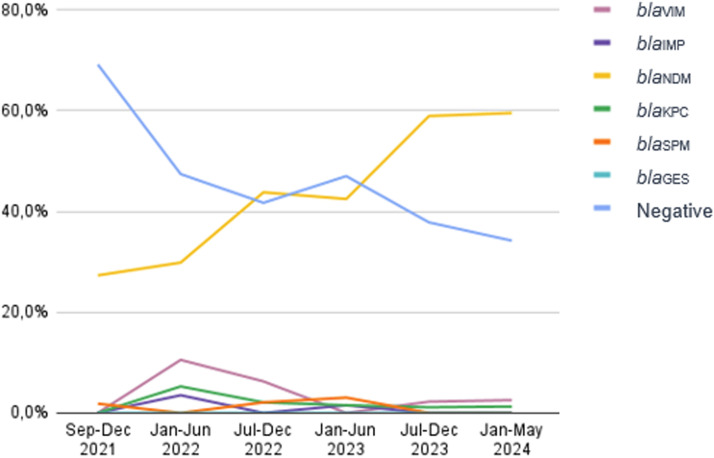
Prevalence of carbapenemase genes in CRPA at Hospital de Clínicas de Porto Alegre ‒ Brazil, from September 2021 to May 2024.

The Brazilian Agency of Sanitary Surveillance issued a document of risk warning [17] for identification of *P. aeruginosa* resistant to carbapenems producing *bla*_KPC_ and *bla*_NDM_ in health care services in Brazil in September 2021 and since then we have been evaluated for carbapenemase genes all CRPA in our institution. Until recently, *bla*_VIM_ and *bla*_IMP_ were the most frequently carbapenemase found in CRPA worldwide, with *bla*_SPM_ maintaining regional spread in Brazil [18,19] This same picture was seen in southern Brazil and in our institution [10,11]

A study conducted in Brazil, between November 2015 and August 2016, evaluated 28 CRPA in hospitalized patients, and the main mechanism involved in carbapenem resistance was due to mutations in porin genes that altered expression levels, with low prevalence of carbapenemases and no *bla*_NDM_ detected [20] Another study, conducted by Kiffer et. al., evaluated the prevalence of carbapenemases in brazilian gram negative pathogens from 2015 to 2022. This study demonstrated a high prevalence of *P. aeruginosa bla*_SPM_ in 2015 (22.5 %) which decreased to around 10 % in 2016‒2019 and to less than 5 % in 2020‒2022. Noteworthy, the study of Kiffer et al., indicated that the *bla*_NDM_, which was very uncommon before 2020, presented a steady increase from 2021 to 2022 (8.8 % and 6.9 %, respectively) [12]

In our study we found a significant increase, from September 2021 to May 2024, in carbapenemase in CRPA, markedly due to the *bla*_NDM_ gene. Conversely, CRPA without carbapenemase gene which accounted for around 70 % in September 2021 decreased significantly to less than 37 % in May 2024. Although we did not evaluate efflux pump and porin expression, our results demonstrate that CRPA in our institution are mostly carbapenemase producers, mainly due to the presence of the *bla*_NDM_ gene.

The data from our study is alarming as the treatment of CRPA *bla*_NDM_ would not respond to the treatment with the new beta lactam/beta lactamase combinations such as ceftazidime/avibactam, which has no activity against metallo enzymes such as NDM [21] Furthermore, while *bla*_SPM_ was usually carried in plasmids not associated with other antimicrobial resistant genes,[18] *bla*_NDM_ was found in plasmids carrying genes that lead to resistance to aminoglycosides and fluoroquinolones [13,14]

This study reinforces the need for surveillance of local epidemiology and early detection of the molecular mechanism of CRPA to avoid the empirical treatment with ceftazidime/avibactam and to improve resources against these infections.

## Conflicts of interest

The authors declare no conflicts of interest
